# Promoting Healthy Ageing in South Africa Through Vaccination of the Elderly

**DOI:** 10.3389/fpubh.2021.635266

**Published:** 2021-04-26

**Authors:** Mncengeli Sibanda, Johanna C. Meyer, Kesentseng J. Mahlaba, Rosemary J. Burnett

**Affiliations:** ^1^Division of Public Health Pharmacy and Management, Sefako Makgatho Health Sciences University, Pretoria, South Africa; ^2^South African Vaccination and Immunisation Centre, Sefako Makgatho Health Sciences University, Pretoria, South Africa; ^3^Department of Virology, Sefako Makgatho Health Sciences University, Pretoria, South Africa

**Keywords:** healthy ageing, influenza, herpes zoster, pneumococcal disease, vaccination, elderly

## Abstract

The World Health Organization estimates that globally, the proportion of people aged ≥60 years will more than double by the year 2050, with the majority of elderly people living in low- and middle-income countries such as South Africa. Population ageing is an impending public health concern, potentially negatively impacting on South Africa's economy and health system if the government does not adequately prepare for this change. Globally, many potential solutions to ensure healthy ageing are being discussed and implemented, including adopting a “life-course” approach to vaccination which includes vaccination of the elderly, since they are at considerable risk of severe morbidity and mortality from vaccine-preventable diseases. While vaccines are considered as one of the greatest tools for preventing childhood infectious disease morbidity and mortality, they are under-utilised in strategies for promoting healthy ageing in South Africa, where only influenza vaccination is available free of charge to the elderly accessing public sector healthcare. Population ageing coupled with the high incidence of vaccine-preventable diseases amongst elderly South Africans, necessitates establishing a comprehensive national policy and guidelines for vaccination of the elderly.

## Introduction

Healthy ageing is defined by the World Health Organization (WHO) as “the process of developing and maintaining the functional ability that enables well-being in older age” (1). The WHO's Decade of Healthy Ageing (2020–2030) was conceptualised in response to a demographic transition occurring in most countries, where the size of the elderly population (i.e., aged ≥60 years) is increasing in number and proportion. Globally, it is projected that the number of elderly persons will more than double from 1 billion in 2019 to 2.1 billion in 2050 (2). The fastest growth is expected in low- and middle-income countries (LMICs) where 79% of the elderly are expected to be living by 2050 (3). The effect of population ageing will be even more pronounced in Africa where the elderly population size is expected to increase from 69 million in 2017 to 226 million by 2050 (3).

Population ageing, resulting from declines in mortality and fertility rates, started in high-income countries (HICs) as far back as the late nineteenth century (1, 3). LMICs have lagged behind because of lack of access to advanced medical and technological interventions, improved sanitation, and food security (3). As access to these life-saving interventions has improved, mortality rates, especially from infectious diseases, have drastically reduced in LMICs, most of which have now entered the demographic transition. For example, the negative impact the human immunodeficiency virus/acquired immunodeficiency syndrome (HIV/AIDS) epidemic had on life expectancy in South Africa (SA) has been offset by the universal roll-out of antiretroviral treatment. This, combined with a decreasing birth rate, is expected to increase the proportion of SA's elderly population from ~8.4% in 2017 to 15.9% by 2050 (3, 4). This demographic transition may negatively impact SA's economy and health system if the government has not adequately prepared for this change (1).

## Global Strategies and Action Plans Promoting Healthy Ageing

The elderly face unique health challenges compared to younger people. For example, non-communicable diseases (NCDs) such as cardiovascular diseases, diabetes, and cancers are a major cause of morbidity and mortality amongst the elderly (1). Although NCDs are increasing in prevalence, infectious diseases are still a major contributor to the burden of disease, especially in LMICs (5). The increased prevalence of comorbidities and severity of diseases in old age will result in a higher demand for healthcare in the future. The Decade of Healthy Ageing (2020–2030), an initiative of the WHO, allows for a collaborative effort between governments and other stakeholders to improve the health and well-being of the elderly (2). Prior to the Decade of Healthy Ageing, policies and action plans such as the United Nations Madrid International Plan of Action on Ageing (6), the WHO's policy framework on active ageing (7) as well as the World Health Assembly resolution WHA69.3 *Global strategy and action plan on ageing and health 2016-2020* (8) were developed. A common theme across these policies and plans is an improvement in the quality of life and achieving the highest possible standard of health for the elderly (1, 2, 6–8). Some of the health-related resolutions include: strengthening NCD policies to promote active ageing; strengthening palliative care as a component of comprehensive care throughout the life course; and ensuring equitable access to healthcare services for the elderly (1, 2, 8). Of note is that vaccination of the elderly is included as a global strategy in all these policies and plans.

## Importance of Vaccines for the Elderly

The rationale behind why vaccination of the elderly is a key strategy for promoting healthy ageing is because the immune system becomes weaker with advancing age through the process of immunosenescence. At a physiological level, there is an age-related defect in the immune system which causes diminished response to antigens by the T-cells and to a lesser extent B-cells (9). Immunosenescence makes the elderly more susceptible to infectious diseases and at increased risk of serious complications, disability, loss of independence, and death (10).

For example, the elderly are at considerable risk of death or disability from pneumonia, which resulted in 6.8 million hospitalisations and 1.1 million deaths globally amongst the elderly in 2015 (11). The leading cause of community acquired pneumonia (CAP) is *Streptococcus pneumoniae* infection, which is a vaccine-preventable disease (VPD). *S. pneumoniae* also causes invasive pneumococcal disease (IPD) (namely pneumococcal meningitis and bacteraemia), with the elderly, especially those with co-morbidities, being at risk of IPD (12). In addition, influenza, the leading viral cause of CAP, disproportionately affects the elderly, who made up 67% of the estimated 294,000–518,000 annual global influenza-related respiratory deaths between 2002 and 2011 (13). Influenza infection may also cause systemic complications including cardio- and cerebrovascular events in some susceptible individuals (14).

Another example is herpes zoster (HZ), a VPD commonly known as shingles, caused by reactivation of latent varicella zoster virus (VZV) due to impaired cell-mediated immunity (e.g., due to immunosenescence or immunosuppression) (15). Primary VZV infection causes chickenpox, a common childhood disease in countries where early childhood varicella vaccination is not offered (15). Following convalescence, VZV remains latent in the sensory nerve ganglia, with the risk of reactivation increasing considerably from the age of 50 (15, 16). In unvaccinated populations, it is estimated that 50% of those aged 85 years will have experienced HZ at least once (16). HZ manifests initially as a painful, unilaterally distributed maculopapular rash which then becomes vesicular and is confined to a single dermatome. Post-herpetic neuralgia (PHN) is a common complication of HZ which is often debilitating and can adversely affect the quality of life of the patient (10, 17). Up to 20% of infected people will experience disturbances of vision or complete blindness (HZ ophthalmicus) (16). There is also an increased risk of vasculopathy following HZ infection which can manifest as stroke, aneurysm, spinal cord infarction, or death if left untreated (16, 18).

Vaccines are recognised as one of the most cost-effective public health measures to prevent infectious diseases, saving an estimated 2.5 million lives annually (19). The benefits of vaccines outweigh any risks and the WHO recommends a “life-course” approach to vaccination (19). Globally, significant strides have been made in strengthening infant vaccination programmes over the past few decades; however, vaccine coverage amongst the elderly remains low (20). The “life-course” approach to vaccination encourages equity in vaccine access so that every age group, including the elderly, benefits from the protection offered by vaccines (19).

Preventing CAP, IPD, and HZ through a vaccination programme specifically targeting the elderly has the potential to significantly promote healthy ageing, and reduce the burden on a country's healthcare system (20). While it is true that immunosenescence results in lower immune responses and reduced vaccine effectiveness in the elderly than in children, vaccines have been proven to play an important role in preventing disease, disability, and premature death amongst the elderly (9). Despite these benefits, vaccines for the elderly were previously generally under-recognised in discussions to promote healthy ageing (20). In contrast, the Decade of Healthy Ageing (2020–2030) emphasises that strengthening of primary healthcare for the elderly must include access to safe, effective and affordable vaccines (2).

The community benefits of vaccination programmes are generally considered in terms of creating herd immunity. However, an added benefit of all vaccination programmes, whether for infants or the elderly, is the reduction of antimicrobial resistance (AMR) (21). Worldwide, AMR is increasingly becoming a public health concern and again the elderly are more at risk of acquiring multidrug-resistant infections and contribute to AMR for a number of reasons (21–23). These include immunosenescence, impaired immune function due to chronic diseases, and other factors such as increased and longer contact with healthcare facilities (due to higher morbidities in old age), staying in old-age homes (which increases the chance of transmission of infectious diseases such as influenza and pneumococcal pneumonia), and the use of medical devices e.g., catheters which are predisposed to bacterial colonisation (21–23). Vaccines are recognised as one of the vital pillars of antimicrobial stewardship, since by preventing VPDs they reduce the demand for antimicrobial treatment (21). Thus, recommendations for vaccination against *S. pneumoniae* and influenza for preventing CAP in the elderly, also aim to reduce antimicrobial usage and AMR (22). For example, it has been estimated that if an influenza vaccine with only 50% effectiveness in the elderly was administered annually to 30% of elderly South Africans, this could avert 390 antimicrobial prescriptions per 100,000 population per year (24). The important role of pneumococcal vaccines in antimicrobial stewardship is highlighted by a decline in the prevalence of penicillin-resistant pneumococcal strains in IPD and nasopharyngeal carriers (25) and indirectly, by a reduction in the antibiotic prescriptions following a decline in pneumococcal disease (26).

## Vaccination Recommendations for the Elderly in High-Income Countries

Many HICs have adopted a “life-course” approach to vaccination and have established recommendations for adult vaccination (27). Specific guidelines are available for special adult populations, including the elderly and those with comorbidities (e.g., HIV infection, end stage renal disease, and diabetes) (28–32). Four vaccines are commonly recommended for the elderly in HICs: influenza vaccine, pneumococcal vaccine, HZ vaccine, and a vaccine combining tetanus toxoid, reduced diphtheria toxoid, and acellular pertussis (TdaP) (28–32).

Influenza vaccination is one of the most cost-effective disease prevention strategies for the elderly (33). Currently, influenza vaccines that are recommended as safe and effective for use in the elderly include an inactivated trivalent vaccine against two strains of influenza A virus (H3N2 and H1N1) and one influenza B virus, and an inactivated quadrivalent vaccine that contains an additional strain of influenza B (33, 34). The quadrivalent vaccine is beneficial for first-time vaccine recipients who may lack immunity to the additional influenza B strain (34). Influenza vaccines are reviewed annually in order to adapt their composition to match the circulating influenza strains determined by surveillance data from the WHO. Hence, annual vaccination against influenza is recommended and should be carried out before the start of the influenza season, for maximal benefit (33, 34).

Vaccines against pneumococcal disease have significantly reduced the burden of IPD and CAP worldwide (35). Presently, two vaccines against *S. pneumoniae* are recommended for the elderly: one dose of 13-valent (against serotypes 1, 3, 4, 5, 6A, 6B, 7F, 9V, 14, 18C, 19A, 19F, and 23F) pneumococcal conjugate vaccine (PCV13), followed at least 1 year later by one dose of 23-valent (against serotypes 1, 2, 3, 4, 5, 6B, 7F, 8, 9N, 9V, 10A, 11A, 12F, 14, 15B,17F, 18C, 19A, 19F, 20, 22F, 23F, 33F) pneumococcal polysaccharide vaccine (PPSV23) (35). Administering a dose of PPSV23 before a dose of PCV13 may reduce the immune response to PCV13, thus vaccine naïve elderly persons should preferably receive PCV13 first, followed by PPSV23 1 year later (35, 36). In a situation where an elderly patient is PCV13 naïve but has already received PPSV23, at least 1 year should pass before administering PCV13 (35, 37). However, in the United States (USA) where herd immunity from sustained high coverage of PCV13 in children has reduced the burden of PCV13-serotype disease in the elderly, the latest Advisory Committee on Immunization Practices (ACIP) guidelines recommend administering one dose of PPSV23 to immunocompetent elderly persons (29, 38). Currently ACIP continues to recommend PCV13 for the PCV13-naïve elderly with an immunocompromising condition, cerebrospinal fluid (CSF) leak, or cochlear implant. For PCV13-naïve immunocompetent elderly persons, PCV13 may be considered for nursing home residents and those living in or traveling to settings where paediatric PCV13 coverage is low (29, 37).

Vaccination is the best public health intervention to prevent HZ and currently two types of vaccines are available for use in adults aged ≥50 years (10, 33, 39). HZ and PHN incidence have been shown to be reduced by 51.3% and 66.5%, respectively, following administration of a single dose of the live attenuated HZ vaccine to adults aged ≥60 (17, 40). This vaccine, licensed since 2006, is contraindicated for the severely immunocompromised elderly, who should rather be vaccinated with the inactivated recombinant vaccine, first licensed in 2017 (28). It has been shown that two doses of the recombinant vaccine, administered 2–6 months apart, elicits a greater and more long-lasting immune response (prevents HZ in 97% and 90%, respectively, of those aged ≥50 and ≥70 years) than the live attenuated vaccine (39, 41, 42). However, local reactions are more common with the recombinant vaccine, and further studies are needed to determine its cost-effectiveness (39).

Finally, a booster dose of the TdaP vaccine is recommended every 10 years, throughout the entire lifetime of all healthy children and adults, because immunity against all three diseases wanes over time (43). Tetanus has a fatality rate of up to 100% if left untreated and infants and the unvaccinated elderly are at high risk of death (43). Recent outbreaks of diphtheria present a public health threat with the potential to reverse the gains made through successful vaccination programmes, with the unvaccinated elderly being at risk (43). Pertussis can cause death especially in unvaccinated and exposed infants aged <2 months, and although there is a potential risk of transmission from unvaccinated elderly persons in close contact with infants under their care, evidence does not support vaccinating the elderly solely for the purpose of “cocooning” their grandchildren (43).

Despite the available guidelines in some HICs and the growing body of evidence supporting the use of vaccines in the elderly, vaccine coverage remains lower than national targets (5, 44). For example, the influenza vaccine coverage amongst the elderly in the USA during 2015 was 73.5%, while only 63.6%, 16.5%, and 34.2% were vaccinated against pneumococcal disease, TdaP, and HZ, respectively (5). The low vaccine coverage may be due to several barriers such as a lack of understanding of the benefits of vaccines by patients and their healthcare providers; limited vaccine efficacy data on elderly populations resulting in low healthcare provider confidence; pervasive societal perceptions that vaccinations are only for children; the lack of “whole-of-life” vaccination records and registers; high costs of vaccines; the lack of comprehensive vaccine guidelines and lack of funding in some HICs which in turn causes a lack of access to vaccines (5, 10, 44).

## South African Recommendations for Vaccination of the Elderly

SA's healthcare system comprises the well-resourced private sector and the poorly-resourced government-run public sector, serving 16% (i.e., those with health insurance) and 84% (i.e., those without health insurance) of the population, respectively (45). Access to quality health services regardless of age, race, gender or socio-economic class is a constitutional right in SA (45); however, socio-economic inequalities and inefficiencies in healthcare delivery have prevented the realisation of this constitutional right (45). Due to resource constraints, only medicines and vaccines listed in the Standard Treatment Guidelines and Essential Medicines List (STGs/EML) are used in the public sector (46). The private sector is more liberal in its medicines offerings and the choice of which medicines are used is usually at the discretion of the prescriber and/or depends on the patient's or healthcare funders' ability to pay (46). The National Health Insurance Bill, released in 2019, aims to facilitate a new revised healthcare system that will ensure equitable access to health services for the entire population in the future (47).

Similar to other LMICs, SA has a high burden of VPDs affecting the elderly (22, 48, 49). For instance, SA has a high burden of CAP caused primarily by influenza and *S. pneumoniae*, with the elderly and those living with HIV being at high risk (22), while influenza and its complications are responsible for >10 million cases and >11,000 deaths annually in SA, with the elderly being most affected (48, 49). Similarly, the incidence of IPD is highest in the elderly, especially those living with HIV (50). Also, while data on the burden of HZ in SA's elderly are lacking, HIV infection is a well-established risk factor for HZ (15, 16), thus, given SA's high HIV prevalence, it is likely that the country's elderly population bears a greater burden of HZ than that experienced by the elderly living in HICs.

There are several vaccines licensed for SA's elderly, including inactivated trivalent and quadrivalent influenza vaccines, PCV13, PPSV23, the live attenuated HZ vaccine, and TdaP. However, the public sector STGs/EML currently only recommends free influenza vaccination for the elderly (51). In addition, the elderly who received PPSV23 for asplenia or CSF leak before reaching 65 years, should receive a free booster at age 65 (51). In contrast, professional bodies such as the South African Thoracic Society and the Federation of Infectious Diseases Societies of Southern Africa, and national agencies such as the National Institute for Communicable Diseases, provide guidelines for vaccination of the elderly, which include PCV13, PPSV23 (22) and influenza vaccines (Table 1) (22, 52). However, PCV13 must be purchased through the private sector, which is prohibitively expensive for most elderly South Africans without health insurance. In addition, the immunocompetent elderly must also purchase the relatively inexpensive PPSV23 through the private sector. While SA private sector recommendations are more comprehensive than the STGs/EML, when compared to HICs (Table 1) there are still several deficiencies (22, 28–31, 52).

**Table 1:** Comparison of recommended vaccines for the elderly in four countries (22, 28–31, 37, 51, 52).

	**Australia**	**USA**	**Germany**	**South Africa**
Influenza vaccine	Annual vaccine	Annual vaccine	Annual vaccine (>60 years)	Annual vaccine (>65 years)
Pneumococcal vaccine	Non-Indigenous Australians: One dose (>70 years) Aboriginal and Torres Strait Islander peoples: Administer a dose of PCV13, followed by first dose of PPSV23 12 months later (2–12 months acceptable), then second dose of PPSV23 at least 5 years later (>50 years)	One dose PPSV23 >65 years >65 years who have immunocompromising condition, CSF leak, cochlear implant: Single dose of PCV13 followed a year later by PPSV23 PCV13 followed a year later by PPSV23 may be considered for >65 years in settings with low pediatric PCV13 coverage e.g. nursing homes	One dose PPSV23 >65 years	≥65 years who are vaccine naive should receive a single dose of PCV13 followed a year later by PPSV23 ≥65 years who have received PPSV23 should receive a single dose of PCV13 at least one year later
HZ vaccine	2 doses recombinant HZ vaccine (>70 years)	2 doses recombinant HZ vaccine (>50 years) or 1 dose live attenuated HZ vaccine (>60 years)	2 doses recombinant HZ vaccine (>50 years) with a minimal and maximum interval of 2–6 months, respectively, between doses.	No recommendation
Tetanus vaccine	Booster dose of a tetanus-containing vaccine recommended for adults ≥50 years who have not received a tetanus-containing vaccine in the past 10 years (but previously completed a primary course of 3 tetanus doses)	Every 10 years	Every 10 years	No recommendation
Reduced strength diphtheria vaccine	One booster dose with tetanus (dT) or booster dose with pertussis vaccinations	Every 10 years	Every 10 years	No recommendation
Acellular pertussis vaccine	Single booster dose for adults who are in close contact with infants (if >10 years since the previous dose)	Once in adulthood	Once in adulthood	No recommendation
Measles vaccine	No recommendation	No recommendation	One dose of measles vaccine for those aged >18 years and born after 1970 with no vaccination or uncertain vaccination history or only one vaccination during childhood	No recommendation

## Conclusions

Since the successful introduction of antiretroviral treatment for HIV/AIDS, SA entered into a demographic transition which is expected to see the elderly comprise almost 16% of the population by 2050. Population ageing is expected to have major negative impacts on SA's health system and economy in the next few decades, unless the WHO global strategy and action plan on healthy ageing (8) is adopted. The South African elderly are particularly prone to increased morbidity and mortality due to VPDs such as CAP, IPD, and HZ. Although vaccines generally have a lower immunogenicity in the elderly, vaccines are still the most cost-effective preventative strategy against infectious diseases, thus it would be prudent for the National Department of Health of SA to establish comprehensive policies and guidelines for vaccination of the elderly. The policies should be accompanied by strategies to address barriers to vaccination of the elderly to create high public demand and uptake. These include educational campaigns targeting the elderly, their caregivers and healthcare providers, emphasising vaccination benefits and effectiveness at preventing severe disease and death in the elderly (5, 10, 53). Also, convenient, easy access to free or affordable vaccination services is essential. Since the elderly often make use of multiple healthcare providers, a universal health record and a whole-of-life vaccine register will help track vaccine use in the elderly (10).

SA's health system is considered as an innovator and pace-setter for many sub-Saharan African countries (54), thus the implementation of such guidelines may have a positive impact on health ageing, not just in SA but in the whole of sub-Saharan Africa.

## Author Contributions

JM, RB, and MS conceptualised the topic and the initial layout of the manuscript. MS wrote the first draft. All authors contributed by collecting related references, writing and reviewing the subsequent versions of the manuscript, read and approved the final version, and take equal responsibility for the contents of the manuscript.

## Conflict of Interest

The South African Vaccination and Immunisation Centre receives unrestricted educational grants from the vaccine industry. The authors declare that the research was conducted in the absence of any commercial or financial relationships that could be construed as a potential conflict of interest.
